# Evaluating the sub-national fidelity of national Initiatives in decentralized health systems: Integrated Primary Health Care Governance in Nigeria

**DOI:** 10.1186/s12913-017-2179-2

**Published:** 2017-03-21

**Authors:** Ejemai Amaize Eboreime, Seye Abimbola, Felix Abrahams Obi, Obinna Ebirim, Olalekan Olubajo, John Eyles, Nonhlanhla Lynette Nxumalo, Faith Nankasa Mambulu

**Affiliations:** 1 Centre for Health Policy, School of Public Health, University of the Witwatersrand, Johannesburg, South Africa; 2 Department of Planning, Research and Statistics, National Primary Healthcare Development Agency, Abuja, Nigeria; 3 University of Sydney, School of Public Health, Edward Ford Building A27, Sydney, NSW 2006 Australia; 4 Health Policy Research Group, College of Medicine, University of Nigeria, Enugu, Nigeria; 5 Direct Consulting and Logistics, Abuja, Nigeria; 6 School of Geography and Earth Sciences, McMaster University, Hamilton, Canada

**Keywords:** Primary healthcare, Implementation fidelity, Decentralization, Nigeria, Health policy

## Abstract

**Background:**

Policy making, translation and implementation in politically and administratively decentralized systems can be challenging. Beyond the mere sub-national acceptance of national initiatives, adherence to policy implementation processes is often poor, particularly in low and middle-income countries. In this study, we explore the implementation fidelity of integrated PHC governance policy in Nigeria’s decentralized governance system and its implications on closing implementation gaps with respect to other top-down health policies and initiatives.

**Methods:**

Having engaged policy makers, we identified 9 core components of the policy (Governance, Legislation, Minimum Service Package, Repositioning, Systems Development, Operational Guidelines, Human Resources, Funding Structure, and Office Establishment). We evaluated the level and pattern of implementation at state level as compared to the national guidelines using a scorecard approach.

**Results:**

Contrary to national government’s assessment of level of compliance, we found that sub-national governments exercised significant discretion with respect to the implementation of core components of the policy. Whereas 35 and 32% of states fully met national criteria for the structural domains of “Office Establishment” and Legislation” respectively, no state was fully compliant to “Human Resource Management” and “Funding” requirements, which are more indicative of functionality. The pattern of implementation suggests that, rather than implementing to improve outcomes, state governments may be more interested in executing low hanging fruits in order to access national incentives.

**Conclusions:**

Our study highlights the importance of evaluating implementation fidelity in providing evidence of implementation gaps towards improving policy execution, particularly in decentralized health systems. This approach will help national policy makers identify more effective ways of supporting lower tiers of governance towards improvement of health systems and outcomes.

**Electronic supplementary material:**

The online version of this article (doi:10.1186/s12913-017-2179-2) contains supplementary material, which is available to authorized users.

## Background

### Introduction

Effective implementation processes are important for achieving the outcomes of complex health system interventions [1] and traditional linear approaches have not been effective in achieving the desired outcomes of such interventions. When evidence-based initiatives are introduced to new settings, they do not automatically get implemented as designed due to differences in context [2]. It is therefore necessary not only to focus on executing the intervention but to also ensure fidelity and quality in implementation. Implementation here refers to “efforts designed to get evidence-based programs or practices of known dimensions into use via effective change strategies” [2]. Given that investments in evidence-based interventions often fail to achieve expected results particularly in low- and middle-income countries (LMICs), there are ongoing debates and discussions on how to evaluate health system interventions to understand the mechanisms and elements that influence their execution and outcomes [3]. Batalden’s observation that “every system is perfectly designed to get the results it gets”, has become associated with improving the implementation of health system strengthening initiatives [4]. To ensure effective implementation, it is important to understand the systems in which interventions are implemented.

Generally, government systems are designed as multi-level structures [5]. While in unitary systems, there is a clear chain of command between national and sub-national levels of government, federal (decentralized) systems are characterized by sharing of authority across the various tiers of government [5]. However, the delineation between both systems is not clear cut, as “unitary” does not indicate that all decision making is done at the national level. Neither do politically decentralized systems confer all decisions-making powers to sub-national government structures. Rather, classification is with regards to the tier at which sovereignty lies [6]. But policy making in unitary systems is largely central, thus implementation can be more efficient than in decentralized systems, given the reduction in bureaucratic bottlenecks (common in decentralized systems) between policymaking and execution [5]. For example, a recent study found that African countries operating federal (decentralised) system of government consistently performed lower in vaccination coverage than the continent’s average [7]. Notably, with the propagation of democracy (particularly in Africa), decentralization is increasingly adopted even in constitutionally centralized countries [6]. Consequently, understanding how this system affects the health policy chain becomes important to ensure optimal benefits of decentralization while minimizing its untoward effects.

Administrative decentralization manifests in three forms: deconcentration, delegation and devolution, [8]. Considered the weakest form of decentralization (and a characteristic of unitary systems), deconcentration redistributes decision-making power across different levels of the central governance authority, by moving its actors from the centre to act as sub-national representatives in regions and/or sub-regions. Delegation, transfers decision-making power from the central authority to semi-autonomous institutions accountable, but not fully subservient to it. Devolution occurs when central government transfers decision-making powers to autonomous sub-national (local) institutions which have independent administrative systems from the central administration. These local governments have legally defined geographical boundaries as well as independent financial management systems. Decentralized (federal) political governance is characterized by devolution of powers [5, 8, 9]. Abimbola et al. described decentralisation in relation to primary health care (PHC) as “a system of governance in which the power, authority, resources, and responsibility for PHC service delivery are transferred from a central government to actors and institutions at the periphery”.

In this paper, we explore the implementation fidelity of integrated PHC governance policy in Nigeria’s decentralized governance system and its implications on closing implementation gaps with respect to other nationally initiated health policies and programmes. Fidelity has been described in 5 dimensions: adherence, exposure, quality of delivery, participant responsiveness, and programme differentiation [3, 10–12]. We focus on the “adherence” dimension that reflect the extent to which policy components are implemented by lower levels of the health system as prescribed by policy guidelines [3, 13].

### Study context

Since independence from British colonial rule in 1960, Nigeria has fluctuated between democratic federal systems, unitary military autocracies and hybrid (a mix of autocratic central government and pseudo-democratic sub-national governments) governance systems [14]. Much of the principles underpinning the current structure of the health system were developed during military and hybrid eras; particularly the devolution of responsibility for health to the various tiers of government. While Nigeria regained democratic rule in 1999, these principles of decentralisation were retained in the 2004 national health policy [15]. Nigeria operates a three-tier federal system of government comprising the federal government, 36 states and 1 territory (the Federal Capital Territory), which in turn consist of Local Government Areas (LGAs) totalling 774 nationally. The states are semi-formally clustered into six geopolitical zones, each with an average of 6 states having comparable sociocultural characteristics, without any administrative structure [16].

While it has led to a considerably decentralized governance system, Nigeria’s constitution is silent on the functions and responsibilities of each tier of government in the provision and oversight of health services [17]. Nevertheless, the National Health Policy prescribes a system in which PHC is under the purview of local governments while the state and federal governments are responsible for the management (administrative and financial) of secondary and tertiary health care services respectively [15]. One of the consequences of PHC being the least resourced (technically and financially) level of government in Nigeria (i.e. Local Governments) is that other levels of government have had to assume a level of responsibility for PHC as well. This system in which functions, structures and human resources for PHC are managed by different tiers and organs of government is poorly coordinated and lacks defined accountability mechanisms. Many services are organised along vertical lines with poor integration and limited co-ordination. Referrals across levels of care are dysfunctional. Diverse management structures co-exist with duplicated or poorly defined roles and responsibilities within and between the three tiers of government [18–20].

One strategy Nigeria adopted for central coordination of the health sector within its decentralized system is the National Council on Health (NCH). The NCH is recognized as the highest policy making body for health in Nigeria, tasked with the responsibility of setting national visions and goals for health to be implemented across the various levels of government. The NCH consists of all state ministries of health, represented by their commissioners, and is chaired by the national minister for health [15, 21]. This composition assumes that decisions taken by the NCH should find easy implementation at sub-national levels, given that the commissioners for health are the highest authority figures for health at the state government level. Another strategy to improve coordination within the health system is the Integrated Primary Health Care Governance initiative, also called “Primary Health Care Under One Roof (PHCUOR)” – the focus of this study. The aim is to improve uniformity in access and quality of care for the majority of the population [22, 23]. This policy was introduced in 2010 by the Federal Ministry of Health through the National Primary Health Care Development Agency (NPHCDA), in response to the challenge of weak governance at the lower levels of the health system [18]. The NCH approved PHCUOR as a national policy in its 54^th^ session, May 2011.

The PHCUOR policy prescribes that a state level management agency, commonly referred to as the State PHC Development Agency (SPHCDA) should be established by each state government to govern all aspects of PHC thus eliminating the problem of fragmented governance. The PHCUOR reforms are hinged on the core principles of “One Management, One Plan, One Monitoring and Evaluation System” thus integrating PHC governance at the state level within an organizational system administered by the SPHCDA. To stimulate compliance to the policy by sub-national governments, the national government included a provision in the 2014 National Health Act known as the Basic Health Care Provision Fund [21]. This fund comprises of not less than 1% of the Consolidated Revenue of the Federation as well as grants from international donors and funds from other sources. States can only access the funds through their respective SPHCDAs after fulfilling requirements stipulated in guidelines developed by the NPHCDA [21]. Many donors now require states to have set up SPHCDAs as a part of requirements for receiving grants for PHC development.

**Fig. 1:**
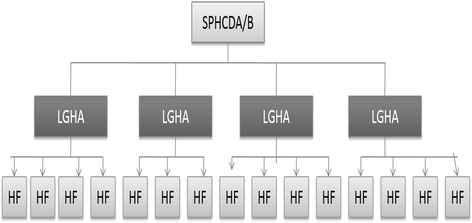
Organizational Structure of Primary Health Care Under One Roof (Source: [18])

In its ideal form, the SPHCDA is expected to absorb all PHC staff who were hitherto employees of the other ministries, departments, and agencies of both state governments and LGAs. The deconcentrated LGA arm of the SPHCDAs is to be known as the Local Government Health Authorities (LGHAs) and should report to the Chief Executive of the SPHCDA (Fig. 1). This is a radical transition from the existing system in which the LGA department of health answers to the LGA Chairman (an elective political position in government). Expected to be autonomous of the State Ministries of Health and to only report to the State Governors through the State Commissioners for Health [18], the SPHCDAs are to be the sole employers of all human resources for PHC, and managers of all PHC-related financial resources, programmes and facilities in a state [18, 19]. The policy is an adaptation of World Health Organization’s District Health System [19, 24].

The government of Nigeria currently reports that 28 out of 37 states “now have State Primary Health Care Development Agencies or equivalent institutions with 26 of them having a legal basis for establishment” [13], thus implying that over 70% of states have complied with the national policy. This assumption, however, does not consider the degree of implementation or operationality of these SPHCDAs as defined by national guidelines. In this study, we evaluate the implementation fidelity of PHCUOR in Nigeria to understand how the intervention was executed and adapted at the sub-national level. While there are many studies on implementation fidelity at the micro (clinical) and meso (organizational) levels of the health system, little has work has been done in evaluating implementation fidelity at the macro level of health systems, particularly as relates to translation of national policies to lower levels.

## Methods

This PHCUOR implementation fidelity assessment was part of a larger health system reform project which also included developing and enacting the National Health Act, and institutionalizing systems level quality improvement through quarterly PHC reviews [18, 21, 23]. To drive the implementation, a team (the National Steering Committee) was set up, consisting of representatives of government (NPHCDA and Federal Ministry of Health) as well as about 30 inter- and non-government organizations including the Nigeria Governors Forum, UNICEF, WHO, CDC, Health Reform Foundation of Nigeria, etc. The team developed implementation guidelines for the implementation of PHCUOR at sub-national levels. In 2013, the NPHCDA finalized the framework and guidelines to ensure implementation harmony across all states. These were validated by key stakeholders represented in the National Steering Committee and approved by the National Council on Health the same year and disseminated to all key policy actors [13]. The PHCUOR guidelines identify 9 domains which must be strengthened at the state level for full implementation [18]. While some aspects of the policy were considered adaptable to context, these 9 domains are considered the core components of the policy which every state was expected to implement (Table 1). A scorecard approach was adopted to evaluate fidelity of state adaptations to the policy with respect to these domains [13].

**Table 1:** Brief description of the PHCUOR framework and Fidelity Assessment Criteria

Domain	Brief Description	Fidelity Assessment Criteria
1. Governance and Ownership	State governments are required to appoint governing bodies and establish organizational leadership structure for the SPHCDAs. The governing body is crucial for setting the PHC vision, winning resources, and holding implementers to account.	Available organogram, appointed chief executive with management team distinct from governing board, accountability mechanism evidenced by periodic report and established reporting lines through the commissioner for health
2. Legislation	The Policy requires that state governments enact laws and regulations for the establishment and functioning of the SPHCDAs. Legislation provides the legal framework while regulations are more specific, containing the enabling language and details of actions needed.	Gazetted law in place having undergone stakeholder consultation, passage by legislature and assent by the governor
3. Minimum Service Package (MSP)	The MSP allows states to classify their facilities according to the adopted system and then determine resource needs for each facility. This approach provides evidence for effective, equitable planning and resource allocation for health	Costed MSP developed, adopted and funded. Health facilities classified and service delivery planning and funding done using the costed MSP
4. Repositioning	Managing organizational change, re-orientation, capacity building and mentoring of managers in the new and old structures to align with new roles and responsibilities	Agency law transfers all PHC structures and functions to the SPHCDA. Stakeholders engaged to create awareness and buy-in to the implications of reforms. Re-orientation plan for staff available and being implemented
5. Systems Development	Establishing state & sub-state structures with one management, one plan, one M&E system; ensuring an appropriate governing board oversees the management team	SPHCDA strategic plan available. Operational plans for state and LGAs available. Financial management policy in place. Guidelines and procedures for recruitment of staff into state and sub-state level structures available. Integrated Supportive Supervision plans implemented quarterly as planned. Clinical guidelines for various interventions available at service delivery level
6. Operational Guidelines	Developing policies, procedures and regulations for HR, procurement/supply chain, accounting/financial management and monitoring & evaluation aligned with national and state policies	PHCUOR implementation guidelines and regulations adapted and operational
7. Human Resources	Effective system for managing human resource issues such as appointment of management staff at state and sub-state levels, addressing mal-distribution of staff, ghost workers and cadre imbalance and plans to train, attract, incentivise and retain staff in unattractive postings.	Committee to implement transfer of all PHC human resources from parallel structures to the Agency established and transfer completed. Human resource audit conducted and operational Human Resource Information System available. Job descriptions available for facility managers and all health workers. Costed Human Resource capacity building plan available. Clear staff recruitment procedures available for all levels of governance
8. Funding Sources and Structure	Developing financial management systems, budget processes, audits, pooled funding and take off-grant	Dedicated budgeting and fund release system for the Agency operational. Integrated PHC (basket) funding system operational. LGA financial contributions deducted at source. Payment of salaries of all health workers at service delivery level, as well as benefits and pensions, fully the responsibility of SPHCDA.
9. Office Establishment	Provision for physical structures, infrastructure and equipment to enable the SPHCDA function	Agency offices established at state and sub-state levels

Sources: [13, 18]

### Implementation scorecard development

The PHCUOR implementation fidelity scorecard was introduced in 2012 to assist states identify, in a systematic manner, areas within the PHCUOR framework in which support may be provided towards effective implementation. The scorecard also serves as peer review mechanism as well as an advocacy tool to the various states and non-governmental stakeholders to facilitate uniform implementation of the policy nationwide [13]. Since 2012, three scorecards (ours being the third) have been developed to monitor the absorption of the policy. We limit the scope of this paper to findings from the third scorecard in 2015, which included an iterative quality improvement to improve the validity and reliability of the scorecard.

### Development of assessment tools

Drawing lessons from strengths and limitations of the earlier two scorecard development processes [13], we sought to develop a scorecard (see Additional file 1) with high validity, reliability and acceptability to stakeholders. We consulted widely with stakeholders and subject matter experts in developing the fidelity assessment tool to gain national consensus and eliminate bias. We conducted a content analysis of the policy document and implementation guidelines, identified key components (Table 1) and developed questionnaires to measure essential milestones in each domain. We also developed semi-structured questionnaires to help explain processes and constraints in implementing the policy (Additional file 2). These tools were distributed to key stakeholders who were later invited to a consensus conference during which the tools were finalized and adopted. Further, we pilot-tested the assessment tools in the Federal Capital Territory to ensure their face and content validity. This paper utilizes findings from the quantitative assessment only.

### Data collection

To ensure quality of the process, we primarily recruited data collectors from the pool of those involved in the initial two assessments of PHCUOR implementation, and remaining gaps were filled by 12 new data collectors. We conducted a two-day training for all data collectors (total of 58). The training included assessment of data collectors’ understanding of the content of the tools. This was done through pre- and post-tests as well as practical demonstrations at plenary sessions thus reducing interviewer bias and checking reliability of the tools. We dispatched data collectors to the states in pairs (one being resident and the other non-resident to the state of assignment). This was done to improve ease of access and while also reducing interviewer bias. Data collection was carried out in all states between 28 August and 5 September, 2015. Prior to the deployment of data collectors, states were notified in writing about the exercise by NPHCDA. The notification explained that this was a national assignment aimed at monitoring implementation of the policy as well as identifying areas in which each state required support, and that feedback will be communicated to them by the National Council on Health.

A list of required items and documents for verification was sent to each state two weeks ahead of the data collection exercise. These were to be provided in either digital format (where available) or hard copies signed by the Chief Executive of the State PHC Agency (SPHCDA), permanent secretary of the State Ministry of Health (SMOH) or their representatives. Semi-structured questionnaires were also dispatched to the states ahead of the field visits. The management team of the SPHCDAs (or SMOH in states yet to establish SPHCDAs) was interviewed as a group (all team members were present together during interviews and consensus was reached on responses). Further, management teams were requested to provide responses on behalf of the state government. A minimum of two weeks was given to each state for consultation. Interviews were only carried out after states indicated readiness by fixing the dates for the interviews. Thus, the responses obtained represented the state government position rather than individual position of interviewees. The teams were instructed to collect evidence for affirmative responses and pay verification visits to three LGAs per state. Verbal affirmative responses without accompanying evidence were recorded as negative responses. We received responses from the health management team of all 36 states and the Federal capital territory.

### Data processing and analysis

An Excel based data-analysis tool was developed for the analysis of the data. Data entry was done independently by two persons, thereafter comparison and harmonization was done to avoid errors. A 4-day evidence review with content analysis of documents received from each state was carried out to validate responses; and 25 analysts drawn from both government and non-governmental interest groups were involved in the data analysis to ensure transparency and reduce bias. Validation process and rules were agreed upon. Analysts were divided into teams tasked to conduct content analysis of the documents. Group findings were subsequently presented at plenary sessions to develop consensus. As a rule, any affirmative answer to the questionnaire which was not backed by documentary evidence was changed to a negative response. And only evidence available at the time of data collection and analysis was accepted. Any progress made by states outside the period of review was excluded from the process. Every positive answer received a score of “1” while negative responses scored “0”.

### Domain weighted averages

To build consensus on the validity of the results as well as assign weights to each of the nine domains, the results of this initial analysis was presented to another group; a group of 9 Subject Matter Experts consisting of high-ranking health sector government policy makers (e.g. directors in the NPHCDA and Federal Ministry of Health), chief executives and senior representatives of selected stakeholder organizations (e.g. Health Reform Foundation of Nigeria, Johns Hopkins University International Vaccine Access Centre and representatives of the National Steering Committee). The weighted averages for each domain were agreed on by these Subject Matter Experts using a modified Nominal Group Technique. We asked each of them to rank each of the 9 domains in order of perceived importance with “1” representing the highest and “9” representing the least important. The results were displayed in plenary with the mean and mode for each domain. Final rankings and weights were assigned through consensus informed by the measures of central tendency. The weighted scores for each state were determined by multiplying domain scores with corresponding weights. Overall score for each state was calculated as the mean of weighted domain scores. The weighted scores for each state is beyond the scope of this paper and discussed in another report [13].

## Results

**Fig. 2:**
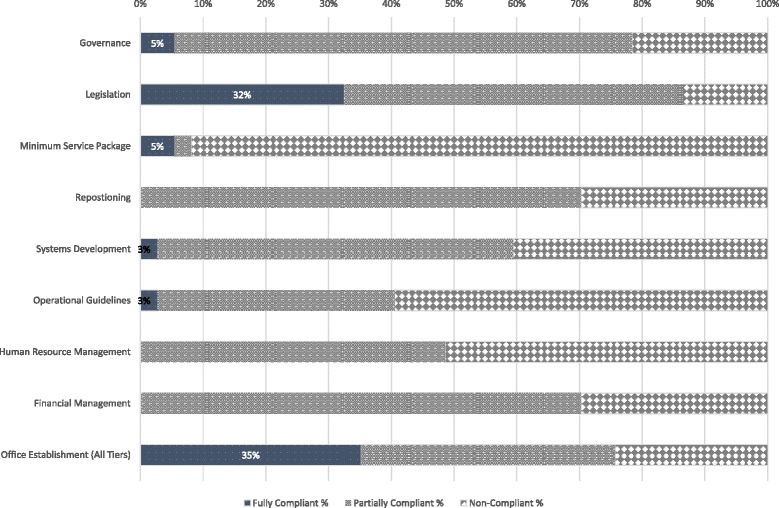
Chart Showing the Level of State Adherence to Each Domain of the PHCUOR Policy

**Fig. 3:**
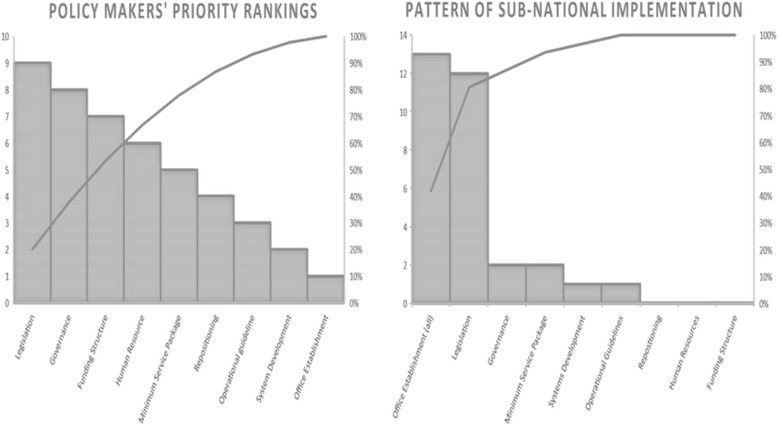
Pareto Charts Comparing National Policy-Makers’ Priority Rankings with Sub-National Implementation Pattern

**Fig. 4:**
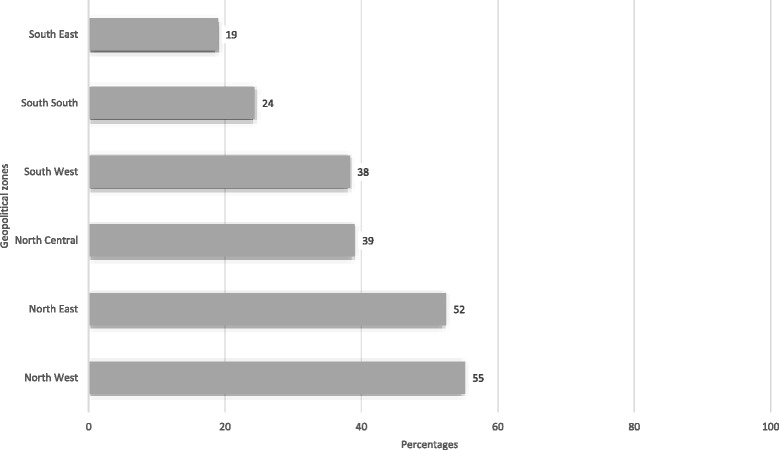
Aggregated Compliance Scores for all domains by Geopolitical Zones (%)

The results from this assessment represent cross-sectional findings from all 37 states and territory (i.e. including the Federal Capital Territory) of Nigeria. Following the criteria spelt out in the policy guidelines, we found 25 (68%) states had met the criteria for having established State Primary Health Care Development Agencies (SPHCDAs) or equivalent structures. No state met all the stipulated criteria for having a functional SPHCDA or PHCUOR system (Fig. 2). Furthermore, Fig. 3 shows that states were disposed to prioritizing domains that were structural, easily verifiable or politically significant (e.g. Acquisition of office space and passage of enabling laws) as opposed to the more functional domains (e.g. Systems Development, Human resources and Financial management). There was a disparity in compliance levels among the six geopolitical zones of Nigeria (Fig. 4), with the northern states more compliant than southern states.

**Fig. 5:**
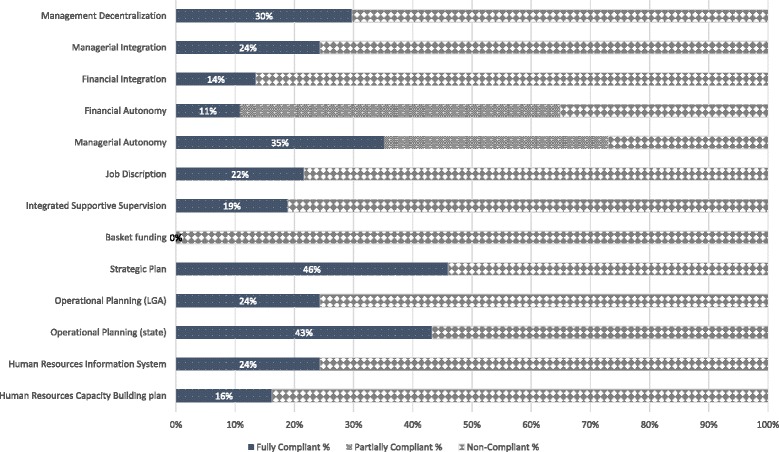
Measures of Elements of Core Principles of PHCUOR

In addition, none of the SPHCDAs adhered completely to the core principles of “One Management, One Plan, One Monitoring and Evaluation System” as the management of PHC was still fragmented across various government agencies. Only 16 (43%) and 9 (24%) states had available operational plans for PHC at the state and LGA levels respectively for the year in reference. No state had a functional integrated operational planning and financing mechanisms for all PHC programmes and interventions. Contrary to the core principles, only 4 (11%) states had financially autonomous SPHCDAs, and 11 (35%) states were managerially autonomous from the State Ministry of Health or other parallel structures. Integration (managerial and financial) of PHC functions was poor, management deconcentration was also weak as only 11(30%) SPHCDAs had functional offices in the LGAs (Fig. 5). In the remainder of the results segment, we present our findings on each of the 9 domains highlighted in Table 1.

### Findings on each of domain

1. **Governance:** Governance was weak in most of the states. Reporting structures were often undefined. Whereas only 15 (60%) SPHCDAs had the roles of the management board being distinct from the governing boards as prescribed by the policy, the others had the Chief Executive of the Agency’s management team doubling as the Chairperson of the governing board. This structure does not appear to allow for transparency and accountability.
2. **Legislation:** Even though 32 (76%) states had passed laws establishing the SPHCDAs, only 12 (32%) of the laws passed through the complete prescribed process (Table 1). Furthermore, the content of the state laws was at significant variance with the principles of PHCUOR in the policy guidelines. The details of this non-compliance are reflected in the findings below with respect to the other domains.
3. **Minimum Service Package (MSP):** The guidelines require all SPHCDAs to develop costed MSP which will guide classification of health facilities according to services provided and basic health needs of the communities served. The document is expected to be an evidence-based resource planning tool for health service provision, particularly about universal (free) access to basic maternal and child health services. Only 2 (5%) states met the criteria for having a functional costed MSP. Thus, health planning remains speculative, rather than evidence-based, and poorly linked to financing services.
4. **Repositioning:** This essentially includes legislative and change management processes required to ensure smooth transition and implementation of the reforms. No state met the guideline’s criteria for repositioning, while 26 (70%) states partially implemented the guidelines, mostly the legislative requirements, but omitting re-orientation, stakeholder engagement and other change management processes.
5. **Operational Guidelines:** These are regulations that guide the functioning of the SPHCDAs (including the departmental structures and relationships as well as clinical guidelines and standard operating procedures. Whereas only one state was found to comply with the all requirements for this domain, 14 (38%) had guidelines which were not in use.
6. **Systems Development:** Only one state met the requirements for full implementation in this domain. Strategic and operational planning is necessary to create systems that can produce expected outcomes. These are further strengthened by regular effective integrated supportive supervision/monitoring and evaluation systems as prescribed by the guidelines. This study found 17 (46%) and 16 (43%) SPHCDAs having strategic plans and operational plans respectively at state level. Only 9 (24%) had operational plans at the LGA level- where service delivery is domiciled (Fig. 5).
7. **Human Resources:** Only 6 (16%) states had capacity building plans for health workers, 9 (24%) had functional Human Resource Information Systems (HRIS) and 8 (22%) had job descriptions available for health workers and managers. Human resource recruitment and management systems were non-existent in 19 (51%) SPHCDAs while no state was fully compliant with human resource requirements prescribed by the guidelines.
8. **Funding Structure:** No state adhered to the prescription of funding structure for PHCUOR. While some states had established a basket funding system, in practice, these systems were either not functional or not integrated (selective for specific vertical programmes). Basket funding requires that all PHC funds from all sources (including development partners) are pooled into one “basket” and distributed to various programmes and components of PHC based on an integrated operational plan adopted by all stakeholders. Dedicated PHC budgeting system for the SPHCDAs was not implemented in most states, and staff salaries and benefits were not under the control of most Agencies. Only 4 (11%) SPHCDAs met these criteria for financial autonomy and poorly integrated financial management systems were observed generally.
9. **Office Establishment:** Operational offices for the SPHCDAs at state and LGA levels are required by the guidelines for effective functioning. While policy makers ranked this as the least important domain, it was the domain with the highest sub-national implementation rate, i.e. 28 (76%) compliant states. However, states concentrated on having offices only at the state capital while neglecting the main operational bases for PHC as only 13(35%) states had offices at the LGAs.

## Discussion

Our findings highlight the challenges of health policy implementation in politically and administratively decentralized systems. Notably, the strategy in place to ensure sub-national implementation fidelity of national health initiatives (the National Council on Health) was not effective in the implementation of PHCUOR. The assumption that decisions ratified at the National Council on Health will translate into policies at the state government level fails to consider that the political system overrides the health administrative system. The implication of this is that the elected state governors are the ultimate decision-makers at that level (the commissioners are political appointees of the governors). The state governors are not under any obligation to adhere to national decisions with respect to health. State governors make policy decisions after conferring with the State Executive Committees (SEC) which comprise of commissioners from all sectors (health included), secretary to the state government as well as the executive governor of the state (who chairs the SEC) and his/her deputy [17].

**Fig. 6:**
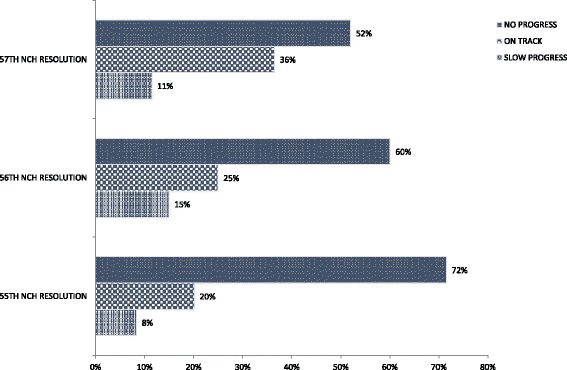
Status of Sub-National Implementation of 55th -57th NCH Resolutions (Source: [25])

Thus, the commissioner for health seeks to convince the SEC to adopt national health policies as a priority in relation to the demands and interests of other sectors. And certain decisions at state level (such as the PHCUOR policy) must be ratified by the state legislature [17], which also needs to be convinced by the SEC of its importance. In the course of these processes, many national initiatives get modified, under-implemented or even rejected. A recent analysis by the Federal Ministry of Health at the 58^th^ NCH revealed that only 20–36% of resolutions in the three consecutive preceding Councils were implemented according to schedule [25]. Fig. 6 further shows progress with implementation rates over the years. The PHCUOR policy was one of such resolutions of the National Council on Health. Over the years, the National Council on Health has reviewed progress with implementation of the policy and passed resolutions aimed at improving implementation at the lower levels.

The strategy to incentivise states to implement PHCUOR – i.e. the National Health Act provision to channel federal funds for PHC through SPHCDAs [21] – also seems to be only partly effective. The number of states with SPHCDAs increased from 16 in 2012 to 25 at the time of this assessment [26]. However, our findings show that this response appears to be superficial as states focus more on implementing domains that relate to structural establishment of SPHCDAs (i.e. enacting enabling laws establishing the SPHCDAs and providing offices at the state capitals) rather than full implementation of the functional aspects of the policy. This response suggests that states may be giving an impression of compliance by fulfilling easily verifiable domains to access federal funds, rather than implementing the reforms out of the need to strengthen PHC. This results in a more complicated governance system than the status quo characterized by conflicts between the newly created SPHCDAs and the existing State Ministries of Health (as both continue to oversee aspects of PHC within the state without a clear delineation of responsibilities) [27].

We also found that implementation fidelity was greater in states in northern geopolitical zones with strong support from donors and development partners or with governors who had shown strong commitment towards improving their health sector performance [13]. These findings are in line with inferences by Okpani and Abimbola (2016) that in Nigeria, differential levels of political support and prioritisation contribute substantially to health services performance in decentralized systems [28]. Our study also reflects, as in a previous study in Nigeria [7], the possible contribution of decentralization to supply-side health disparities across states, with implications on sustainability of health reforms in Nigeria (given the autonomy and discretionary power of states). Implementing and sustaining health system initiatives in decentralized systems require evolution of strategies that gain full sub-national political commitment. Whereas central government’s direct intervention at lower levels may have “quick win” effects, long-term sustainability has been poor [26, 28–30]. Thus, in addition to the suggestion that central coordination may be necessary for equity of outcomes in decentralized health systems [31], our findings suggest that central government role in top-down initiatives in federal settings requires sustained advocacy and incentives to facilitate the effective transfer of responsibility to lower levels of government where the power of implementation resides.

Sabatier and Mazmanian suggest that for national policies to be effectively implemented at sub-national levels, the policies must have clear and logically consistent objectives; there should be an adequate causal theory as to how specific activities would lead to desired outcomes; implementation process must be structured to ensure adherence by implementers (such as incentives and sanctions); committed and adequately skilled implementing officials must be available; support from interest groups and legislature; and no changes in socio-economic situations that undermine political support or the causal theory fundamental to the policy [32]. Hogwood and Gunn developed a more demanding list of preconditions for ‘perfect implementation’. Some of these preconditions include availability of adequate time and sufficient resources as well as required combination of these resources. Other requirements are that the relationship between cause and effect must be direct and that policy makers can demand and obtain perfect compliance [33].

Given that these conditions are not likely to all be present at the same time [5], particularly in LMICs where finance and human resource constraints are common, complete implementation may not be expected. For example, in Uganda (low income economy), financial constraints at the lower levels of its administratively decentralized health system is a constraint on the implementation capacity of the lower levels of governance [9, 34]. Human resource for health challenges in South Africa (a politically decentralized upper middle income country) has similarly been found to affect implementation of national initiatives designed to reduce health inequalities. Nevertheless, strategies can be put in place to optimise implementation.

Based on our findings, we recommend the following strategies to improve sub-national implementation fidelity in politically decentralized systems such as Nigeria (although the complexity of health systems makes it difficult to guarantee maximum implementation fidelity in any system, not least decentralized ones):

1. Prioritize operational and implementation research (including stakeholder analysis) as a part of the policy process to provide evidence for advocacy to policy makers and implementers at all levels of governance. Such research should include implementation comparative fidelity assessments to promote peer competition among sub-national level decision makers and implementers towards improving compliance and ensuring quality implementation.
2. Ensure consistent advocacy to all stakeholders (particularly decision makers) on the relevance and benefits of the policy. Particularly identify and engage power structures (final decision-making authorities), e.g. state governors through the Nigerian Governors’ Forum in the Nigerian context, as this has shown to be effective in improving sub-national acceptance and implementation of national initiatives [13, 25].
3. Provide incentives, supportive supervision and mentoring to lower decision-making levels with respect to policy implementation and change management. These have been shown to facilitate sub-national implementation of some NCH approved initiatives in Nigeria.
4. Although direct national level intervention at the lower levels have shown some effect in stimulating absorption of some previous national policies in Nigeria, this strategy should be used sparingly as this results in over-dependence by states on the national government to implement what should otherwise be the responsibility of the lower tiers. Examples of this effect in Nigeria are the Midwives Service Scheme, Primary Health Care Reviews and the SURE-P MCH interventions [26, 28–30, 35].

Limitations in this study include its cross-sectional approach to examine an ongoing policy process. This approach potentially obscures some of the events that facilitated implementation of the policy which may have been brought to light if longitudinal implementation patterns were examined from conception to implementation, and if detailed qualitative exploration of sub-national implementation was conducted. However, our intention is not to evaluate the PHCUOR policy in isolation but to elicit lessons from its implementation to inform and improve other similar policy processes. Comparative analysis of findings from all three scorecards, including methodological variations, are detailed in another report [13]. Another potential limitation is that we attempt to apply recommendations from one policy process to other contexts. But this is not uncommon in health systems research on complex interventions and processes as insights obtained in one setting are often transferable to other settings [36].

## Conclusions

Our study highlights how evaluating implementation fidelity can provide evidence of gaps in implementation, thereby potentially improving policy execution, particularly in decentralized health systems. Using this approach will help national policy makers identify more effective ways of supporting lower tiers of governance to improve health systems and outcomes. Further, evaluating implementation fidelity can guide policy stakeholders at all levels in evidence-based advocacy and other strategies to ensure effective policy execution.

## Additional files

Additional file 1: Scorecard data collection tool (PDF 541 kb)

Additional file 2: Semi-structured questionnaire (PDF 200 kb)

## Abbreviations

HRIS: Human Resource Information System
LGA: Local Government Area/Authority
LMIC: Low and middle income countries
MSP: Minimum service package
NCH: National Council on Health
NPHCDA: National Primary Health Care Development Agency
PHC: Primary health care
PHCUOR: Primary health care under one roof
SEC: State Executive Council
SMOH: State Ministry of Health
SPHCDA: State Primary Health Care Development Agency
SURE-P MCH: Subsidy reinvestment programme for maternal and child health
