# Supplementary material for: Evaluating the sub-national fidelity of national Initiatives in decentralized health systems: Integrated Primary Health Care Governance in Nigeria
Source: BMC Health Serv Res. 2017 Mar 21;17:227. doi: 10.1186/s12913-017-2179-2 (PMC5361827; doi:10.1186/s12913-017-2179-2)
Supplement: Supplementary file 1 — Scorecard data collection tool (PDF 541 kb) [file 12913_2017_2179_MOESM1_ESM.pdf]

NATIONAL PRIMARY HEALTH CARE DEVELOPMENT AGENCY

Plot 681 /682 Port Harcourt Crescent, off Gimbiya Street,
Area 11, Garki, Abuja.

REVISED TOOL FOR ASSESSMENT OF ESTABLISHMENT AND FUNCTIONALITY OF STATE PRIMARY HEALTH CARE DEVELOPMENT AGENCIES/BOARDS

**State:**					
**Organisation:**					**Date:**
**S/N**	**1.0. GOVERNANCE AND OWNERSHIP**	**Yes**	**No**	**Sources of Data**	**Notes**
1.1	Is there a physical structure (building) called the SPHCDA/B or its equivalent?			SPHCDA, SMOH	Sight it and take pictures
1.2	Is there an organogram for the SPHCDA/B?			SPHCDA, SMOH	Sight and obtain copy
1.3	Is there an appointed head of the SPHCDA/B? Name Official designation (ES, ED, EC?)			SPHCDA, SMOH	
1.4	If "Yes" to Q1.3 above, does the head of the SPHCDA/B report to the Executive Governor through the Hon. Commissioner of Health?			SPHCDA, SMOH	
1.6	Does the SPHCDA/B hold top management meetings at least once a month?			SPHCDA, SMOH	Sight and obtain copy of minutes
1.7	Is there a SPHCDA/B Governing Board?			SPHCDA, SMOH	
1.8	Is there a document specifying the role of the Governing Board as distinct from the role of the Management Team (SPHCDA/B)?			SPHCDA, SMOH	Sight and obtain copy

1.9	Does the SPHCDA/B publish monthly, quarterly or annual reports as part of accountability mechanisms?			SPHCDA, SMOH	Sight and obtain copy
	Total				
	Percentage Score = (Total Yes/total Number of Questions*100)				
**S/N**	**2.0. LEGISLATION**	**Yes**	**No**	**Sources of Data**	**Notes**
2.1	Has the State established a technical committee for the drafting of the Bill to establish the SPHCDA/B?			SMOH, SPHCDA, SMOJ	Obtain copy of team composition
2.2	If "Yes" to Q 2.1 above, has the technical committee been engaging with stakeholders to build consensus on the key elements of the Bill?			SMOH, SPHCDA, SMOJ	Sight and obtain minutes of meeting / report
2.3	Has your State drafted a PHC Bill to establish the SPHCDA/B?			SMOH, SPHCDA, SMOJ	Sight and obtain copy
2.4	Has the Bill reached the State House of Assembly from the Executive Arm of Government?			SMOH, SPHCDA, SMOJ	
2.5	Has a PHC Bill been passed by the State House of Assembly?			SMOH, SPHCDA, SMOJ	
2.6	Has the Governor assented to the PHC Bill passed by the Legislature?			SMOH, SPHCDA, SMOJ	Sight and obtain copy of the law
2.7	Has your State drafted Regulations for operationalizing the Bill when passed into Law?			SMOH, SPHCDA, SMOJ	Sight and obtain copy of the regulations
2.8	Has the Regulation been signed by the Governor or Commissioner as the case may be?			SMOH, SPHCDA, SMOJ	
2.9	Has the PHC Law establishing the SPHCDA/B and Regulations been gazetted?			SMOH, SPHCDA, SMOJ	Sight & obtain copy of gazetted law & regulations

2.10	Are the regulations consistent with the law establishing the SPHCDA/B?			SMOH, SPHCDA, SMOJ	
	Total				
	Percentage Score = (Total Yes/total Number of Questions*100)				
**S/N**	**3.0. MINIMUM SERVICE PACKAGE (MSP)**	**Yes**	**No**	**Sources of Data**	**Notes**
3.1	Has your State adopted a Minimum Service Package for different facility types?			SMOH, SPHCDA	Sight & obtain copy of State MSP
3.2	Has the SPHCDA classified the health facilities in the state based on the MSP?			SMOH, SPHCDA	
3.3	Is the MSP available and being used at the health facility level?			SMOH, SPHCDA, HF sampling	
3.5	Has the MSP been costed?			SMOH, SPHCDA	Confirm in copy of MSP
3.6	Is there funding for operationalizing the MSP for effective and efficient delivery of services?			SMOH, SPHCDA	
3.7	Does your State monitoring team regularly (at least yearly) evaluate the resource gaps for implementing the MSP?			SMOH, SPHCDA	Sight & obtain copy of report or other available evidence
3.8	Do you think the MSP has improved efficiency/work output in the PHC facilities?			SMOH, SPHCDA	
3.9	Is the state implementing any special health care project such as free MCH services, Conditional Cash Transfer for MCH services etc?			SMOH, SPHCDA	Sight & obtain copy of concept note, implementation report or any other evidence

3.10	Is the delivery of the special health care project linked with the costed MSP?			SMOH, SPHCDA	
	Total				
	Percentage Score = (Total Yes/total Number of Questions*100)				
**S/N**	**4.0 REPOSITIONING**	**Yes**	**No**	**Sources of Data**	**Notes**
4.1	Does the Law establishing the SPHCDA/B clearly transfer all PHC functions from the SMOH, MOLG, LGSC and LGA to the SPHCDA/B?			SMOH, SPHCDA, MOLG, LGSC, LGA, SMOJ	Sight, consult & obtain copy of law. Verify at the LGA
4.3	Has there been any forum for engaging with different stakeholders (SMOH, MOLG, LGSC, LGA, Devt Partners, CSOs, Professional bodies, Media etc) to discuss the changing roles and responsibilities as the SPHCDA/B is established?			SMOH, SPHCDA, MOLG, LGSC, LGA	Sight and obtain minutes of meeting / report. Verify at the LGA
4.4	Has the department of PHC at the SMOH been collapsed into the SPHCDA/B?			SMOH	Sight new organogram
4.5	Has the department of PHC at the MOLG been collapsed into the SPHCDA/B?			MOLG	Sight new organogram. Verify at the LGA
4.6	Has the department of PHC at the LGSC been collapsed into the SPHCDA/B?			LGSC	Sight new organogram. Verify at the LGA
4.7	Has the department of PHC in the Local Governments been collapsed into the SPHCDA/B as part of the Local Government Health Authority?			LGA	Sight new organogram. Verify at the LGA
4.8	Is there a plan for the re-orientation of different categories of SPHCDA/B staff?			SPHCDA	Sight & obtain copy of plan

4.9	Using the plan, has any re-orientation activity taken place for SPHCDA staff?			SPHCDA	Sight & obtain copy of report
4.10	Do you think the on-going repositioning process has resolved a gap in the running of the PHC system in your state?			SPHCDA	
	Total				
	Percentage Score = (Total Yes/total Number of Questions*100)				
**S/N**	**5.0. SYSTEMS DEVELOPMENT**	**Yes**	**No**	**Sources of Data**	**Notes**
5.1	Has the SPHCDA/B developed a Strategic Health Plan (usually for 3-5 years)?			SPHCDA	Sight & obtain copy
5.2	Does the SPHCDA/B have bi annual Operational Plan for the current year?			SPHCDA	Sight & obtain copy
5.3	Does the SPHCDA/B have other specific financial management policies separate from the State Civil Service financial regulations to guide its programme activities?			SPHCDA	
5.4	Does the SPHCDA/B have an Integrated Supportive Supervision plan?			SPHCDA	Sight & obtain copy
5.5	Is the SPHCDA/B's Integrated Supportive Supervision plan being implemented?			SPHCDA	Sight & obtain copy of last 3 reports
5.6	Is there an Integrated Supportive Supervision tool?			SPHCDA	Sight & obtain copy
5.7	If "YES", is it used during Integrated Supportive Supervisory visits to LGAs and health facilities?			SPHCDA	Sight & obtain copy of report of previous ISS visit & check for data analysis

5.8	Does the state consistently conduct Integrated Supportive Supervision visits on a quarterly basis?			SPHCDA	Sight & obtain copy of last 3 reports
5.9	Are there guidelines and procedures for recruitment into the SPHCDA/B and sub-State level structures?			SPHCDA	See any documented evidence
5.10	Does the institutional structure of the SPHCDA clearly show lines of accountability?			SPHCDA	See Organogram
5.11	Are there guidelines and protocols for operations at different levels e.g. Standing Orders in PHC facilities; Programme Guidelines (Immunization, Reproductive Health, MCH etc)?			SPHCDA, HF	Sight & obtain copy. Verify at a HF in the LGA
5.12	Is there an Operational Health Plan in the LGAs?			SPHCDA, LGAs	Sight & obtain copy. Verify at a HF in the LGA
	Total				
	Percentage Score = (Total Yes/total Number of Questions*100)				
**S/N**	**6.0. OPERATIONAL GUIDELINES**	**Yes**	**No**	**Sources of Data**	**Notes**
6.1	Has the State adapted the Implementation Manual on PHCUOR?			SMOH, SPHCDA	Sight & obtain copy
6.2	Is the implementation manual in use?			SMOH, SPHCDA	
6.3	Does the State policy on PHCUOR make provision for HR, M&E, Accounting and other procedures to follow?			SMOH, SPHCDA	
6.4	Does the SPHCDA/B have the capacity to develop and implement its work plan independent of the SMOH?			SPHCDA, SMOH	
6.5	Have key personnel (management team) been trained on the mandate of the SPHCDA using the policy guidelines?			SMOH, SPHCDA	Sight and obtain copy of training

					report or attendance list
	Total				
	Percentage Score = (Total Yes/total Number of Questions*100)				
**S/N**	**7.0. HUMAN RESOURCES**	**Yes**	**No**	**Sources of Data**	**Notes**
7.1	Has your State established a high level Human Resource Committee for documentation and transfer of PHC human resources?			SMOH, SPHCDA	See list of committee members
7.3	Has the PHC staff audit, development of database and other related activities been carried out?			SMOH, SPHCDA	Sight and obtain copy
7.5	Has an orientation been organized on Human Resource Information System and MSP for the HR Committee members?			SMOH, SPHCDA	Sight & obtain copy of report
7.6	Are all the staff providing PHC services especially at the health facility level, employees of the SPHCDA/B?			SMOH, SPHCDA, HF	Verify at a HF in the LGA
7.7	Is there an implementation plan for managing issues related to mal-distribution of staff?			SMOH, SPHCDA	Sight and obtain copy of plan, Note actions taken
7.8	Has your State developed Job Descriptions for health facility managers and workers?			SMOH, SPHCDA	Sight & obtain copy
7.9	Are there clear procedures for recruitment of staff for sub-state structures (Zonal and LGA levels)?			SMOH, SPHCDA	Sight & obtain copy
7.10	Is there a costed capacity building plan to address staff needs?			SMOH, SPHCDA	Sight & obtain copy
	Total				
	Percentage Score = (Total Yes/total Number of Questions*100)				

S/N	8.0. FUNDING SOURCES & STRUCTURE	Yes	No	Sources of Data	Notes
8.1	Did your State release a take-off grant for the SPHCDA/B?			SMOH, SPHCDA	
8.2	Is there an established SPHCDA/B dedicated budget process and fund release for planned PHC expenditure?			SPHCDA	
8.3	Is there a system that tracks funds released to the SPHCDA/B?			SMOH, SPHCDA	
8.4	Has the SPHCDB/A developed mechanisms for joint (Basket or Pool) funding for implementing PHC programmes and services in line with the MSP?			SPHCDA, SMOF	Sight & obtain copy of guiding document
8.5	Is the SPHCDA/B able to effectively plan and budget for its activities without external assistance?			SPHCDA	
8.6	Is the SPHCA/B able to plan for procurement of commodities and other items required at the health facility for effective service delivery?			SPHCDA	
8.7	Are the funds allocated to the SPHCDA/B commensurate with its approved plan?			SPHCDA	
8.9	Is the financial contribution by the LGA deducted from source?			SPHCDA, SMOLG, SMOF	
8.10	Are the salaries of health workers at the facility level paid by the SPHCDA/B?			SPHCDA	Verify at a HF in the LGA
8.11	Does the SPHCDA/B administer staff benefits and pension?			SPHCDA	
	Total				
	Percentage Score = (Total Yes/total Number of Questions*100)				
**S/N**	**9.0. Office Set-up**	**Yes**	**No**	**Sources of Data**	**Notes**
9.1	Has your State identified and designated a specific office for the operations of the SPHCDA/B at the state level?			SPHCDA	

9.2	If "Yes" to Q9.1 above, is the office being used by the SPHCDA/B?				
9.3	Has your State identified and designated specific offices for the operations of the LGHAs at the LGA level?				Verify at the LGA
9.4	If "Yes" to Q9.3 above, are these offices being used by the LGHAs?			SPHCDA	Verify at the LGA
9.5	Is the office complex of the SPHCDA/B furnished with office equipment and installations such as furniture, internet, computers and access			SPHCDA	
9.6	Was/Is there a costed start-up plan for the take-off of the management team of the SPHCDB/A?			SPHCDA	Sight document
	Total				
	Percentage Score = (Total Yes/total Number of Questions*100)				

Name of Interviewer 1:	Name of Interviewer 2:
Phone no:	Phone no:
Signature :	Signature :
Date:	Date:
