# Supplementary material for: Evaluating the sub-national fidelity of national Initiatives in decentralized health systems: Integrated Primary Health Care Governance in Nigeria
Source: BMC Health Serv Res. 2017 Mar 21;17:227. doi: 10.1186/s12913-017-2179-2 (PMC5361827; doi:10.1186/s12913-017-2179-2)
Supplement: Supplementary file 2 — Semi-structured questionnaire (PDF 200 kb) [file 12913_2017_2179_MOESM2_ESM.pdf]

NATIONAL PRIMARY HEALTH CARE DEVELOPMENT AGENCY
Plot 681 /682 Port Harcourt Crescent, off Gimbiya Street,
Area 11, Garki, Abuja.

PHCUOR IMPLEMENTATION QUESTIONAIRE

Instructions:

1. *These qualitative questions should be answered by respondents prior to the day of assessment.*
2. *Respondents should include, but are not limited to: the executive secretary or chairman of SPHCDA/B and the management team members.*
3. *Respondents in states without SPHCDA/B would include the commissioner for health or permanent secretary, director PHC, director PRS, state immunization officer and other directors in the state ministry of health.*
4. *Response should be type-written and two copies handed over to the interviewer on the day of assessment.*

Questions:

1. *What are the names, phone numbers and corresponding designations of respondents (Answer in a tabular format as indicated below)?*

S/n	*Name*	*Designation*	*Phone number*	*Signature*

2. *Which year did you introduce your state's SPHCDA/B (or its alternative)?*
3. *How was SPHCDA/B introduced in your state?*
4. *What name do you call your state's SPHCDA/B (write in full)?*
5. *What are the names of the current state's SPHCDA executive director and Board chairman? (if none, why?)*
6. *What is the coordinating platform that is driving the implementation of PHCUOR in your state, who leads it, who is in it and how often do they meet?*
7. *Outline the PHC strengthening processes or activities that have taken place since the last PHCUOR assessment in mid-2012.*
8. *List the partners or organisations in your state that support PHC strengthening processes/activities or PHUOUR implementation (Answer in a tabular format as indicated below).*

S/n	*Partner or organisation*	*Programs*	*Impact on PHC or PHCUOR*
